# Electrospinning Fabricating Au/TiO_2_ Network-like Nanofibers as Visible Light Activated Photocatalyst

**DOI:** 10.1038/s41598-019-44422-w

**Published:** 2019-05-29

**Authors:** Zhuojun Duan, Yingzhou Huang, Dingke Zhang, Shijian Chen

**Affiliations:** 1 Chongqing Key Laboratory of Soft Condensed Matter Physics and Smart Materials, College of Physics, Chongqing University, No.55 Daxuecheng South Rd, Shapingba, Chongqing, 401331 China; 2 School of Physics and Electronic Engineering, Chongqing Normal University, Chongqing, 401331 People’s Republic of China

**Keywords:** Electronic and spintronic devices, Sensors and biosensors

## Abstract

Exploiting photocatalysts with characteristics of low cost, high reactivity and easy recovery offer great potentials for complete elimination of toxic chemicals and environmental remediation. In this work, Au/TiO_2_ network-like nanofibers were fabricated using a facile electrospinning technique followed by calcinations in air. Photocatalytic tests indicate that the Au/TiO_2_ network-like nanofibers possess an excellent photodegradation rate of rhodamine B (RB) under UV, visible and natural light radiation. The enhanced photocatalytic activity can be attributed to the plasmonic resonance absorption of Au nanoparticles, and photogenerated electrons and holes are effectively separated by the Au/TiO_2_ heterojunction structures. Furthermore, the three-dimensional network structure can provide a large number of active sites for RB degradation.

## Introduction

Because of the heavy use of fossil fuels, environmental pollutions, such as air and water pollution, have caused wide public concerns. Photocatalysis, as a “environment-friendly” technology, shows tremendous potentials for complete degradation of toxic contaminant and is identified as one of the most efficient and preiswert means to solve the issue of environmental pollution^1,2^. As a photocatalyst, titanium dioxide (TiO_2_: 3.2 eV for anatase and 3.0 eV for rutile) has the characteristics of inexpensive, nontoxic, environmental friendly and high-efficiency and it has been extensively studied and applied for the elimination of environment pollutants^3–5^. However, TiO_2_ has many drawbacks, such as very wide band gap, fast recombination rate of photogenerated electrons and holes, which hinder the utilization and commercialization of TiO_2_^6,7^. The hole-migration occurs typically through charge transferring from adjacent position, and electrons usually travel faster in the conduction band^8,9^. If electrons and holes are not recombined at once, they can move to the surface of the particle and at there react with chemical species. Therefore, how to improve the separation of photoinduced electron-hole pairs in TiO_2_ is an important issue for exploiting the TiO_2_-based photocatalysts for further applications. Furthermore, due to the wide band gap, TiO_2_ is only activated under UV light. Much attempts have been devoted to exploring TiO_2_ as visible-light-active photocatalysts for effectively utilizing solar energy, which included doping with metal or non-metal elements^10,11^, introducing defects^12^, and coupling with metallic or semiconducting nanoparticles^13–15^.

Recently, noble metals have been testified to do good job in prohibiting electron-hole recombination due to the fact that noble metals would facilitate the separation of photogenerated electron–hole pairs and promote interfacial charge transfer^16–20^. In addition, noble metal nanoparticles have the ability to improve the visible-light absorption of TiO_2_^21–25^. In particular, gold nanoparticles embedded in a TiO_2_ film (Au-TiO_2_) has attracted much attention due to that Au is a virulent precious metal with prominent catalytic activity^26,27^.

Currently, photocatalysts are normally in particle form which are suffering from aggregating. To achieve a high degree of utilization of photocatalyst, dispersing catalysts onto support materials with large surface area are often employed. Electrospinning is a cost-efficient and simple means, it is capable of fabricating network-like three-dimensional (3D) nanofibers that have been studied in many fields because of high specific surface area and mesoporous structure^28–30^. The nanofibers are fabricated by electrospinning that can be successfully prepared as network-like three-dimensional (3D) nanostructures with good stability characteristics^31^. What’s more, because of their particular properties compared with other nanostructure, network-like nanofibers are always attractive. The nanofibers have good thermal stability and excellent charge carrier mobility, which are especially useful to improve the catalytic performance of materials^32–34^. Furthermore, the network-like 3D nanofibers can work as supporters for Au nanoparticles which avoids the problem of aggregating^35^.

For these concerns above mentioned, in this work, we fabricated nanofibers composed of Au NPs and TiO_2_ by electrospinning method. The prepared nanofibers presented a macropores 3D network-like structure. The elementary composition and morphology of Au/TiO_2_ nanocomposite were investigated by X-ray diffraction (XRD) and transmission electron microscopy (TEM). The as-prepared 3D network-like nanofibers showed good photocatalytic performance in the degrading RB under visible and UV light because of the efficient charge separation between the formed heterojunction between Au and TiO_2_ NPs and high efficient utilization of photocatalysts. Besides, because of the long length of the nanofibers, the photocatalysts can be simply recycled by centrifuging, washing and drying with little damage of the photocatalytic performance.

## Experimental Section

### Chemicals and materials

All chemical reagents used in this study were analytical grade without further purification. Tetrabutyl titanate (C_16_H_36_O_4_Ti) and acid glacial (C_2_H_4_O_2_) were purchased from Chengdu Kelon Chemical Reagent Factory. Polyvinylpyrrolidone (PVP) was purchased from Shanghai Macklin Biochemical Co., Ltd. Ethanol (CH_3_CH_2_OH) was purchased from Chongqing Sichuan East Chemical Co., Ltd. Gold chloride solution (HAuCl_4_) and sodium citrate (C_6_H_5_Na_3_O_7_·2H_2_O) were purchased from Shanghai Aladdin biochemical technology Co., Ltd.

### Preparation of Au nanoparticles

The synthetic method is that 2 mL of 50 mM HAuCl_4_ and 98 mL of deionized water were added in flask. The flask was heated in oil bath at 120 °C until the solution boiling. Then, 10 mL of 38.3 mM sodium citrate was added in flask quickly. Finally, keeping heating for 20 min under magnetic stirring, gold nanoparticles were synthesized. In the whole experiment process, the mouth of flask covered a layer of plastic wrap for reducing the evaporation of the water.

### Preparation of Au/TiO_2_ nanofibers

In this paper, Au/TiO_2_ nanofibers were prepared by using electrostatic spinning. The electrostatic spinning set-up was composed by an injector, a spinneret (a 15-gauge stainless steel needle), a high voltage power supply (18 kV), and a grounded aluminum foil used as collector. The precursor solution was consisted of tetrabutyl titanate (1 mL), acetic acid (1.5 mL), ethanol (5 mL) and polyvinylpyrrolidone (PVP) solution (10 mL). The as-prepared precursor solution was stirred at room temperature for about 12 h vigorously. Subsequently, the as-synthesized Au NPs solution with the amount of 5 μL, 10 μL, and 15 μL were added to the precursor solution, respectively. The precursor solution was stirred for 30 min at room temperature after adding Au. Then this mixture was put in a plastic syringe with a 15-gauge spinneret. The whole device was carried out under an electric voltage of 18 kV and was controlled the distance between the tip of the needle and the collector was 15 cm. The injection rate was set at 1.0 mL/h during electrospinning. The obtained fibers were then transferred into the muffle furnace for annealing at 500 °C for 12 h in air with a heating rate of 5 °C/min to produce Au/nanofibers. The as-prepared Au/TiO_2_ nanofiber with different Au NPs contents were marked as Au(x)/TiO_2_, where X represented the volume of milliliters of the Au solution that was added to the precursors solution. For comparison, pure TiO_2_ nanofibers were also made by the same process without adding Au NPs to the precursor.

### Characterization

X-ray diffraction (XRD) analysis for the crystal structures of the samples was executed by X-ray diffractometer (Rigaku D/MAX2500PC) with Cu Kα radiation. The morphologies and size of the samples were tested by scanning electron microscopy (SEM, TESCAN MIRA) and transmission electron microscopy (TEM, FEI TECNAI G2 F20). UV-vis diffuse reflectance spectra of the samples were measured by a UV3600 spectrophotometer (Shimadzu, Japan) and BaSO_4_ was used as a reference.

### Photocatalytic test

The photocatalytic performance of these Au/TiO_2_ nanofibers were measured by detecting the quantities of the RB degradation in water. An internal 350 W Mercury lamp was adopted as UV light source and a 300 W Xe lamp was equipped with an optical filter as visible light (λ > 420 nm). To cool the lamps, a circulating water system was used. Firstly, the initial concentration of the 50 mL RB solution was 10 mg L^−1^ and solid catalyst (0.02 g) was added and the solution was stirred without UV-Vis light for 30 min to obtain a good dispersion for establishing adsorption-desorption balance between the catalyst surface and the organic molecules. At given intervals of irradiation (time interval was 5 min in UV irradiation and 30 min in visible light), the reaction solutions (3 ml) as samples were extracted and centrifuged. Then the filtrates were analyzed by a spectrophotometer to calculated the decreased amount in the concentration of RB solution.

## Results and Discussion

### Structure and morphology

**Figure 1:**
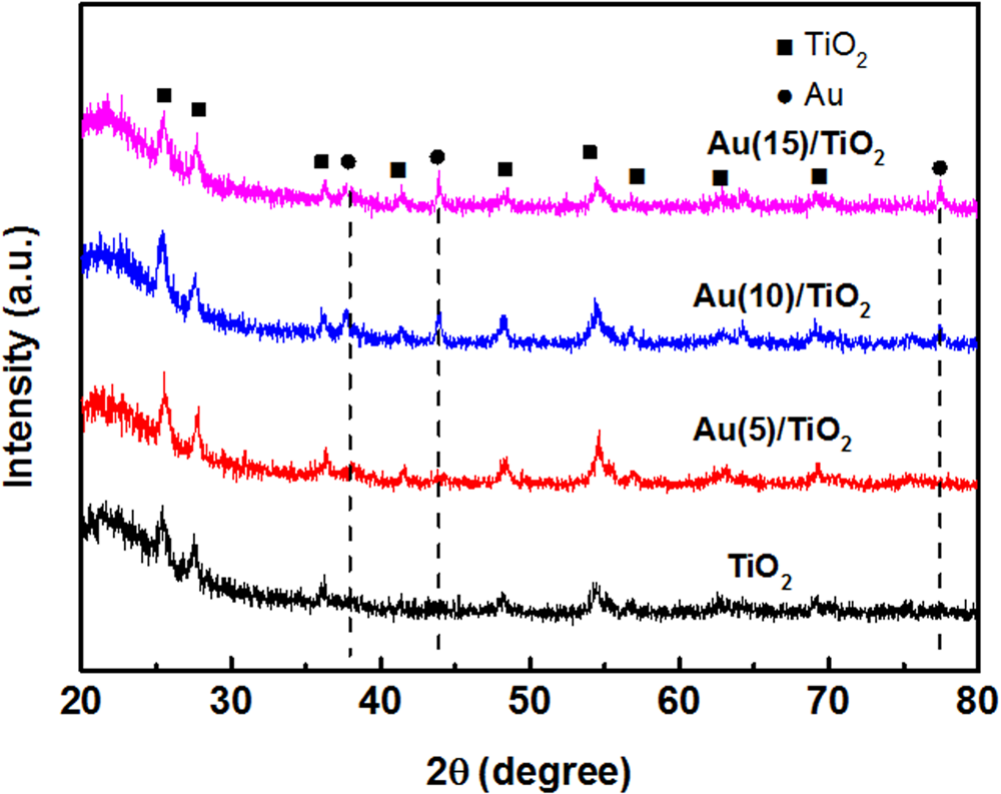
XRD patterns of the pure TiO_2_ nanofibers and Au/TiO_2_ nanofibers Au(5)/TiO_2_, Au(10)/TiO_2_ and Au(15)/TiO_2_.

For revealing the crystalline and phase structures of the prepared nanocomposites, XRD patterns were measured and shown in Fig. 1. Specifically, the peaks at 25.5°, 37.9°, 48.2°, and 55.0° are attributed to (101), (004), (200) and (211) planes, respectively, which is indexed to anatase TiO_2_ (PDF card 21−1272, JCPDS). Besides, peaks at about 2θ = 27.5°, 41.3°, 56.8°, 62.9°, and 69.2° are also observed, which are attributed to the (110), (111), (220), (002) and (301) crystal faces of rutile TiO_2_, respectively. After the Au NPs solution was added to the TiO_2_ precursor, as shown in samples Au(5)/TiO_2_, Au(10)/TiO_2_ and Au(15)/TiO_2_, additional diffraction peaks with 2θ values of 38.2°, 44.3°, and 77.9° appeared, which are corresponding to (111), (200) and (331) crystal planes of cubic Au, respectively (PDF card 04–0784, JCPDS). The above XRD results show that the prepared samples are composites of Au and TiO_2_.

**Figure 2:**
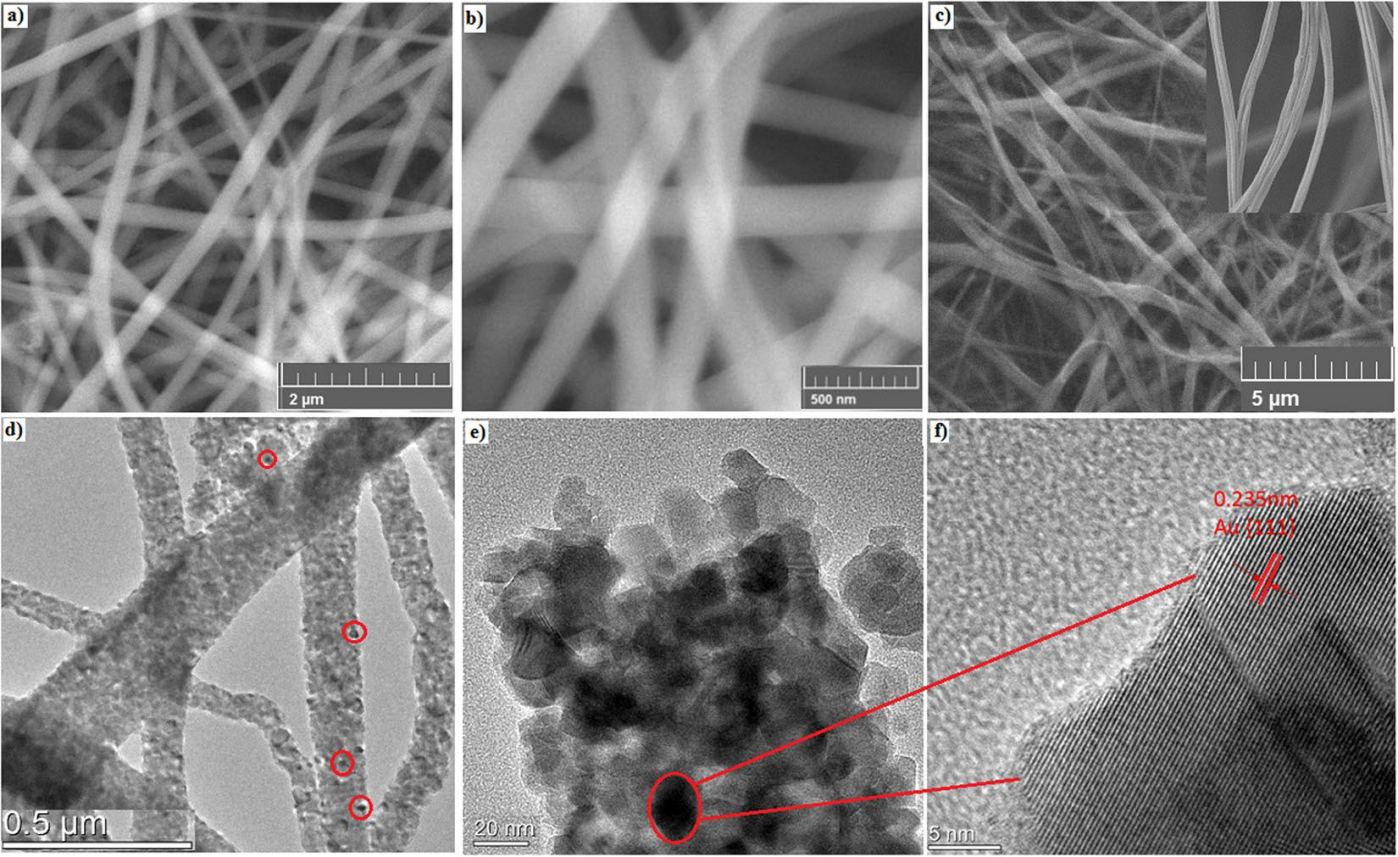
(**a**,**b**) SEM images of pure TiO_2_ with different magnifications; (**c**) SEM images of the sample Au(10)/TiO_2_, inset of (**c**) shows higher magnification; (**d**,**e**) TEM images of the sample Au(10)/TiO_2_ with different magnifications; (**f**) HRTEM image of the sample Au(10)/TiO_2_.

The morphologies of the as-prepared nanofibers were measured by SEM and shown in Fig. 2. Figure 2a shows that the pure TiO_2_ fibers are randomly aligned with the diameter ranging from 100 to 200 nm, and the randomly orientated nanofibers form a 3D network with macropores. SEM image (Fig. 2b) with higher magnification reveals that these TiO_2_ nanofibers have a glossy and homogeneous surface. After adding Au nanoparticles, the Au/TiO_2_ composite remained as a randomly aligned fiber network-like morphology, as shown in Fig. 2c. Because of the small size and low concentration of Au NPs, the surface of Au/TiO_2_ nanofibers remained unchanged (inset of Fig. 2c).

For purpose of further studying the microstructure of the Au/TiO_2_ nanofibers, the TEM and HRTEM images were measured. The low magnification TEM image of the Au/TiO_2_ samples also shows network nanofibers inlaid with particles (Fig. 2d). Meanwhile, a high-resolution image of the Au/TiO_2_ fiber (Fig. 2e) indicates that the nanofibers are composed of many granular particles in size of 10–20 nm. The lattice spacing of 0.235 nm is observed on the HRTEM image (Fig. 2f), corresponding to the (111) planes of Au, which indicates Au nanoparticles were successfully embedded with TiO_2_ particles.

### UV-vis diffuse reflectance spectra

**Figure 3:**
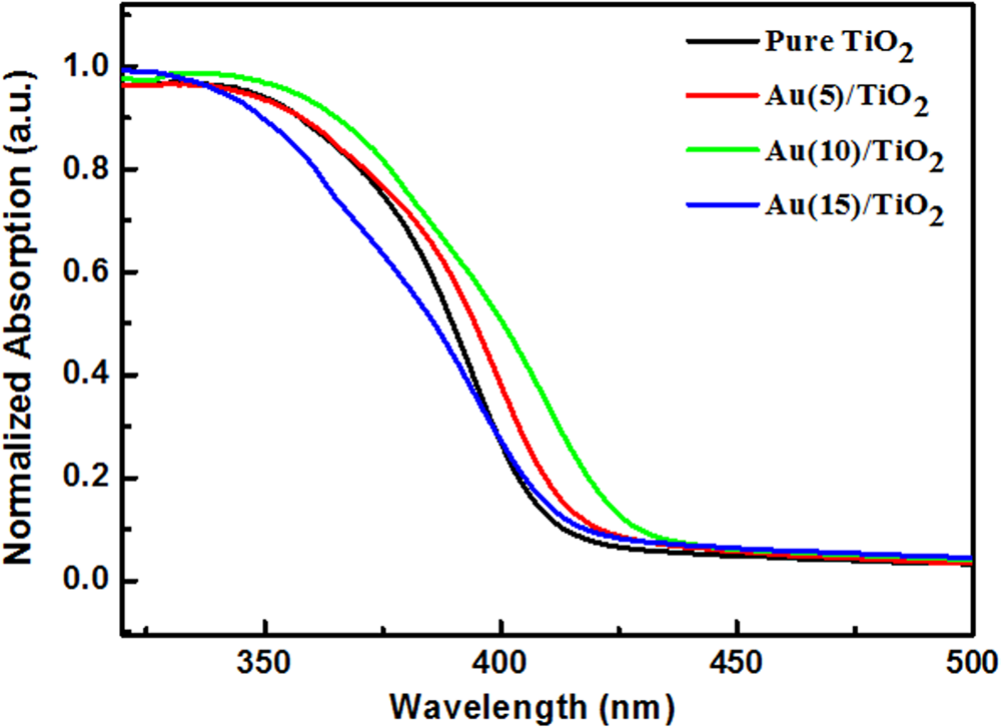
Normalized UV-Vis diffuse reflectance spectra of the pure TiO_2_ nanofibers, Au(5)/TiO_2_ nanofibers, Au(10)/TiO_2_, Au(15)/TiO_2_ nanofibers.

Figure 3 indicates the UV-Vis absorption spectra of the pure TiO_2_, Au(5)/TiO_2_, Au(10)/TiO_2_, Au(15)/TiO_2_ nanofibers. It can be observed that the pure TiO_2_ nanofiber has a sharp absorption edge at 420 nm, which is corresponding to TiO_2_ band gap excitation. The Au/TiO_2_ nanofibers were fabricated by incorporation of as-prepared Au nanoparticles during the electrospinning of TiO_2_ nanofibers and subsequent calcination in air. The main problem in this method maybe lead to the uneven distribution of Au nanoparticles and the Au nanoparticles were embedded in the TiO_2_ nanofibers, which showed no obvious absorption peak from Au nanoparticles in Au/TiO_2_ nanofibers. Furthermore, as shown in Table 1, the actual Au contents of Au(x)/TiO_2_ nanofibers is not high, which maybe lead to no obvious absorption peak as well.

**Table 1:** The actual Au contents of Au(x)/TiO_2_ nanofibers.

samples	Au%
Au(5)/TiO_2_	1.50
Au(10)/TiO_2_	3.60
Au(15)/TiO_2_	5.20

However, the absorption edges of Au/TiO_2_ nanofibers show slightly red-shift, comparing with pure TiO_2_ nanofiber, which indicates an enhanced light absorption for Au/TiO_2_ nanofibers through incorporation of Au nanoparticles.

### Photocatalytic activity

**Figure 4:**
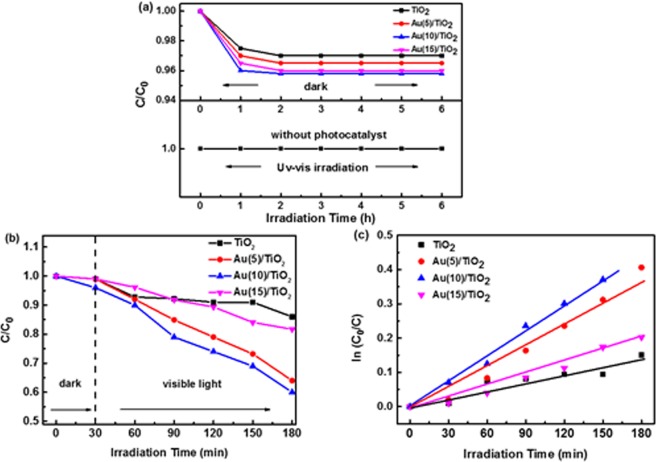
(**a**) Photodegradation rate of RB in the presence of the pure TiO_2_ nanofibers, Au(5)/TiO_2_ nanofibers, Au(10)/TiO_2_ and Au(15)/TiO_2_ nanofibers in the absence of the nanofiber photocatalysts in the dark and with UV-vis light irradiation; (**b**) Photodegradation rate of RB for different nanofibers under visible light irradiation (300 mw/cm^2^); (**c**) Kinetic linear simulation curves of RB degradation under visible light for different nanofibers.

The photocatalytic performance of these Au/TiO_2_ nanofibers were measured by detecting the quantity of the RB degradation in water and the degradation effect of the Au/TiO_2_ nanofiber catalysts was labeled as C/C_0_, where C and C_0_ were marked the remainder and initial concentration of RB, respectively. The pure TiO_2_ nanofibers were acted as a photocatalytic performance reference. As shown in Fig. 4, The control experimental designs of different conditions were as follows: (1) the RB solution with photocatalysts without UV-Vis light irradiation; (2) the RB solution with UV-Vis light irradiation without photocatalysts; (3) the RB solution with photocatalysts with UV light irradiation; (4) the RB solution with photocatalysts with visible light irradiation. After 6 h without UV-Vis light irradiation or without nanofiber photocatalysts, the results (Fig. 4a) showed that there is barely degradations of RB.

TiO_2_ is well known as a very efficient UV light active photocatalyst. In this work, we firstly examined the photocatalytic performance of our prepared TiO_2_ and Au/TiO_2_ nanofibers under UV light irradiation of 100 mw/cm^2^. As expected, all the TiO_2_ and Au/TiO_2_ nanofibers exhibited good photocatalytic activity for degrading RB solution. The TiO_2_ nanofibers could degrade almost 100% RB solution in 30 min under UV irradiation, and Au/TiO_2_ nanofibers even exhibited faster degrading rate than pure TiO_2_ nanofibers. It is worth mentioning that this degrading rate of our Au/TiO_2_ nanofibers can surpass most of the reported TiO_2_ and other UV-activated photocatalysts, as listed in Table 2.

**Table 2:** Comparison of photocatalytic performance of Au/TiO_2_ with other UV-activated photocatalysts under UV irradiation.

Catalyst	Degradation time (min)	photocatalyst dosage (mg)	Power (W)	Dye concentration (mg/L)	Ref.
Au/TiO_2_	30	20	100	10	*This work*
Ag_2_O/TiO_2_	60	9	300	10	*Appl. Surf. Sci. 396 (2017) 530–538*
SnO_2_/TiO_2_	40	25	175	10	*AIP. Conference Proceedings 030029 (2018) 1–5*
CeO_2_/TiO_2_	60	100	250	10	*J. Mater. Sci-Mater. El. 27 (2016) 825–833*
N/Fe/TiO_2_	80	100	250	10	*J. Nanomater. 2016 (2016) 1–11*
P/TiO_2_	50	50	100	10	*J. Colloid Interf. Sci. 516 (2018) 215–223*
AlON/TiO_2_	120	69	300	10	*Ceram. Int. 45 (2019) 6767–6773*
Ni/TiO_2_	120	20	300	10	*Ceram. Int. 40 (2014) 3887–3893*

Exploring TiO_2_ as a visible light activated photocatalyst is of great importance for potential applications. We further evaluated the photocatalytic activity of our prepared nanofibers under visible light irradiation of 300 mw/cm^2^. Not surprising, pure TiO_2_ nanofibers showed a bad degradation rate in visible light, with only degradation rate 5% in 120 min, as shown in Fig. 4b. However, Au/TiO_2_ nanofiber photocatalysts exhibited much enhanced photocatalytic activity in visible light, and the degradation effect of RB was about 35, 42 and 9% after 120 min for the sample of Au(5)/TiO_2_, Au(10)/TiO_2_ and Au(15)/TiO_2_ nanofibers, respectively. The kinetic analysis of degradation of RB which illustrates the photocatalytic efficiency was also evaluated. Because of the initial concentration of RB solution was low (C_0_ = 10 mg/L) in our experiment, and the kinetics linear emulation curve of the photocatalytic performance of these Au/TiO_2_ nanofibers followed the first order kinetics model of Langmuir-Hinshelwood. The explanation is depicted below^36^:

1 $$\mathrm{ln}\,{C}_{0}/C=kKt={k}_{{app}}t$$

where C means the concentration of RB after the reaction (mg/L), t means ultraviolet or visible light illumination time, k means the reaction velocity constant (mg/(L min^−1^)), K means the adsorption index of the reactant (L/mg) and K_app_ means the apparent first-order rate constant (min^−1^). The determined K_app_ for four catalysts is summarized in Fig. 4c. It is revealed that the photocatalytic performances followed the order: Au(10)/TiO_2_ nanofibers >Au(5)/TiO_2_ nanofibers >Au(15)/TiO_2_ nanofibers >TiO_2_ nanofibers.

**Figure 5:**
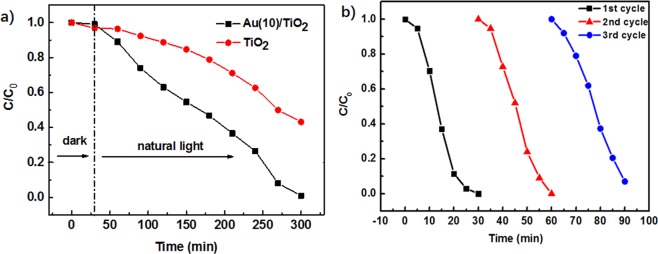
(**a**) Photodegradation rate of RB solution for pure TiO_2_ nanofibers and Au(10)/TiO_2_ nanofibers under nature light irradiation (15mw/cm^2^); (**b**) Photocatalytic activity of the Au(10)/TiO_2_ nanofibers for RB solution degradation with three times of cycling uses.

The photocatalytic activity of the as-prepared pure TiO_2_ and Au(10)/TiO_2_ nanofibers were further compared by RB degradation under natural light irradiation (15 mw/cm^2^). It can be seen that the Au(10)/TiO_2_ photocatalysts exhibited superior photocatalytic performance for degrading RB solution compared with the pure TiO_2_ nanofibers. As shown in Fig. 5a, the degradations of RB by using Au(10)/TiO_2_ nanofibers as photocatalysts reached almost 100% in 270 min irradiation. In the same condition, the degradation of RB by using pure TiO_2_ nanofibers was just 40%.

Stability is an important factor of the catalyst in the practical application. In order to test the stability of the Au(10)/TiO_2_ nanofibers, three recycling experiments were carried under identical conditions. As shown in Fig. 5b, after a three cycle experiment in UV irradiation, the photocatalytic degradation efficiency barely changes, with the degradation rate of about 93% during the third experiment.

### Possible mechanism on the photocatalytic activity

**Figure 6:**
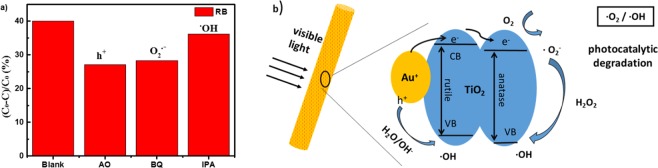
(**a**) Effect of a series of scavengers on the degradation efficiency of RB for Au(10)/TiO2 nanofibers. Blank, Ammonium Oxalate (AO), Benzoquinine (BQ), Isopropanol (IPA) (Illumination time t = 150 min). (**b**) The photodegradation mechanism schematic illustration of the Au/TiO_2_ nanofibers.

As is well known that the main reaction substances include hole (h^+^), hydroxyl radical (•OH) and superoxide radical (•O_2_^−^) in the process of photocatalytic oxidation. For differentiating the role of reactive oxygen species in RB degradation and explaining the reaction mechanism, ammonium oxalate (AO), isopropanol (IPA) and benzoquinone (BQ) were chosen as quenching agents for h^+^, •OH and •O_2_^−^, respectively^37–39^. The experimental results (Fig. 6a) revealed that with the addition of AO and BQ, the photodegradation efficiency of RB decreases from 40% to 27.0% and 28.3%, implying that hole (h^+^) and •O_2_^−^ act as the main reactive oxygen species in the process of photodegradation.

Based on the aforementioned experimental results, a feasible scheme (Fig. 6b) is proposed. In our case, the Au/TiO_2_ nanofibers were irradiated by UV and visible light, respectively. As shown in Scheme 1 for the situation under visible light irradiation, photo-generated electron-hole pairs are appeared at Au NPs because of surface plasmon resonance (SPR)^40^. The conduction band energy of TiO_2_ is lower that the Femi level of Au, but the sprayed electrons coming from gold can transfer to the conduction band of TiO_2_. In our work, The TiO_2_ nanofibers have anatase/rutile phases. O_2_ reduction by the photo-induced electrons on the rutile surface is inefficient but the anatase is more active for O_2_ reduction. As a result, the electrons prefer transfer from rutile to anatase can effectively suppresses the recombination of photogenerated electron-hole pairs and accelerates the photodegradation procedure^41^. The photogenerated electrons transfer to the rutile of TiO_2_ and further transfer to anatase to initiate reacting with the dissolved oxygen and the holes (·Au^+^) react with H_2_O or OH^−^, avoiding the recombination of electron-hole pairs, which can enhance the photocatalytic effect of TiO_2_. The migration of photogenerated electrons is very fast on TiO_2_, also indicating that the photogenerated electron-hole pairs can be effectively separated^42^. In other words, the combination of gold and TiO_2_ is supposed to generate a charge separation condition with relatively mild oxidation (positive gold) and same reduction (TiO_2_ conduction band) potentials as TiO_2_^43^. The process of RB degradation can be further illustrated as following: the photo-induced electrons are injected into the TiO_2_ conduction band (Eqs (2) and (3)). The electrons can combine with the dissolved oxygen molecules and produce •O_2_^−^ (Eq. (4)), then the HOO• are produced by protonation (Eq. (5)), the HOO• and captured electrons react to generate H_2_O_2_ (Eq. (6)), and finally •OH are produced (Eq. (7)). At the same time, the h^+^ can combine with OH^−^ or H_2_O in the solution to form •OH (Eqs (8) and (9)). The RB solution was degraded by •O_2_^−^ and •OH to CO_2_, H_2_O and other environmental pollutants^44^ ((Eqs (10) and (11)).

2 $${Au}+{hv}\to {Au}^{\ast }$$

3 $${{Au}}^{\ast }+{Ti}{{O}}_{2}\to {h}^{+}(Au)+{e}^{-}({Ti}{{O}}_{2})$$

4 $${e}^{-}({Ti}{{O}}_{2})+{O}_{2}\to {Ti}{{O}}_{2}+\bullet {{O}_{2}}^{-}$$

5 $$\bullet {{O}_{2}}^{-}+{H}^{+}\to HOO\,\bullet $$

6 $$HOO\,\bullet +{e}^{-}(Ti{O}_{2})+{H}^{+}\to {H}_{2}{O}_{2}+Ti{O}_{2}$$

7 $${H}_{2}{O}_{2}+{e}^{-}(Ti{O}_{2})\to \bullet OH+O{H}^{-}+Ti{O}_{2}$$

8 $${h}^{+}(Au)+{H}_{2}O\to Au+{H}^{+}+\bullet OH$$

9 $${h}^{+}(Au)+O{H}^{-}\to Au+\bullet OH$$

10 $${Dye}+\bullet {{O}_{2}}^{-}\to C{O}_{2}+{H}_{2}O$$

11 $${Dye}+\bullet OH\to C{O}_{2}+{H}_{2}O$$

## Conclusion

In summary, using a facile electrospinning method followed by calcinations, Au/TiO_2_ nanofibers were successfully fabricated. The nanofibers presented a network-like three-dimensional (3D) structures with macropores and Au particles with a size of 10–20 nm were well dispersed on the TiO_2_ fibers. The prepared Au/TiO_2_ nanofibers exhibited much enhanced photocatalytic activity by degradation of RB under UV, Vis and natural light irradiation. It is believed that the enhanced photocatalytic performance is due to the high utilization of Au particles on the fibers with a three-dimensional network structures which worked as a framework for providing high available Au active sites for degrading RB, and to the efficient charge separation through Au/TiO_2_ heterojunction structure. This study highlights the potential use of electrospinning technique to fabricate TiO_2_ nanofibers as noble metal supports for photcatalysis.

## Data Availability

The authors declare that data in our manuscript are available.
